# Expression patterns, regulatory interactions, and diagnostic potential of LINC00839 and LINC01605 in esophageal cancer

**DOI:** 10.1016/j.bbrep.2025.102305

**Published:** 2025-10-14

**Authors:** Mahdi Bahmani, Ashkan Kalantary-Charvadeh, Morvarid Hamrahjoo, Nasrin Ziamajidi, Roghayeh Abbasalipourkabir, Shayan Marhamati

**Affiliations:** a Department of Clinical Biochemistry, Faculty of Medicine, Hamadan University of Medical Sciences, Hamadan, Iran; b Department of Virology, Faculty of Medicine, Hamadan University of Medical Sciences, Hamadan, Iran; c Research Center for Molecular Medicine, Institute of Cancer, Avicenna Health Research Institute, Hamadan University of Medical Sciences, Hamadan, Iran

**Keywords:** Esophageal Neoplasm, LINC00839, LINC01605, ceRNA network, Diagnosis

## Abstract

Esophageal cancer (EC) is an aggressive malignancy with a poor prognosis. lncRNAs are crucial in EC, but the roles of LINC00839 and LINC01605 are unclear. This study explores their involvement in EC to better understand their potential carcinogenic functions. In this study, the GEPIA database was used to investigate gene expression changes in EC. Next, the DIANA-LncBase tool and the multiMiR package in R software were used to obtain the interaction between the lncRNA-miRNA-mRNA axis. The interactions were constructed in a network using Cytoscape software. Pathway enrichment was performed using the clusterProfiler package. Gene-disease association was examined using the DisGeNET platform. RT-qPCR was used to measure the expression levels of LINC00839 and LINC01605 in EC samples. Finally, ROC analysis evaluated their diagnostic potential. Analysis using the GEPIA database revealed a significant increase in the expression of LINC01605 in EC (log_2_FC = 2.5, P-value<0.05), while the increase in LINC00839 was non-significant. RT-qPCR validation in 18 pairs of EC patient tissues confirmed these findings. ROC curve analysis showed that LINC01605 had significant diagnostic potential (AUC = 0.734), while LINC00839 did not. LINC01605 and LINC00839 were predicted to interact with miRNAs, including miR-16-5p and miR-195-5p. Furthermore, these miRNAs interact with oncogenic mRNAs such as FGF2 and BCL2. GO and KEGG enrichment analyses revealed their involvement in pathways related to protein localization, histone binding, and various cancer types.

LINC01605 exhibits potential as an EC biomarker, actively regulating carcinogenesis through the ceRNA network, whereas LINC00839 demonstrates a comparatively diminished role in EC diagnosis, sharing common regulatory components.

## Highlights

• LINC01605 significantly upregulated in EC; shows diagnostic potential.
• LINC00839 expression is increased in EC, but without statistical significance.
• MiR-16-5p and miR-195-5p interact with both LINC00839 and LINC01605.
• FGF2, MCFD2, and PIK3R1 are shared mRNA targets with approved drugs.
• ROC curve shows LINC01605 is a promising biomarker for EC diagnosis.

## Abbreviations

AUC: Area Under the Curve
BP: Biological Process
CC: Cellular Compartment
ceRNA: Competing Endogenous RNA
DisGeNET: Disease Gene Network
EAC: Esophageal Adenocarcinoma
EC: Esophageal Cancer
EGFR: Epidermal Growth Factor Receptor
ESCA: Esophageal Carcinoma
ESCC: Esophageal Squamous Cell Carcinoma
FGF2: Fibroblast Growth Factor 2
GEPIA: Gene Expression Profiling Interactive Analysis
GO: Gene Ontology
GTEx: Genotype-Tissue Expression
HR: Hazard Ratio
KEGG: Kyoto Encyclopedia of Genes and Genomes
lncRNA: Long Non-Coding RNA
logFC: Logarithm of Fold Change
MCFD2: Multiple Coagulation Factor Deficiency Protein 2
MF: Molecular Function
miRNA: MicroRNA
mTOR: Mammalian Target of Rapamycin
NF-κB: Nuclear Factor Kappa-Light-Chain-Enhancer of Activated B Cells
PIK3R1: Phosphoinositide-3-Kinase Regulatory Subunit 1
PPI: Protein-Protein Interaction
ROC: Receiver Operating Characteristic
RT-qPCR: Quantitative Reverse Transcription Polymerase Chain Reaction
TCGA: The Cancer Genome Atlas

## 1 Introduction

Despite advances in treatment, esophageal cancer (EC) remains one of the deadliest malignancies worldwide [1]. EC is histologically classified into two major subtypes: esophageal squamous cell carcinoma (ESCC) and esophageal adenocarcinoma (EAC) [2]. Current treatment strategies for EC include surgical resection, often complemented by adjuvant therapies such as immunotherapy, chemotherapy, and radiotherapy [3]. However, EC is frequently diagnosed at advanced stages, contributing to its association with distant metastasis and local tissue invasion. As a result, the 5-year survival rate for EC patients remains low, ranging from 15 % to 25 % [4].

Given the poor prognosis and limited effectiveness of current treatment strategies, a growing need exists to better understand the molecular mechanisms underlying EC, including the potential role of regulatory noncoding RNAs. Genome-wide association studies have identified multiple genetic loci significantly associated with the risk of developing EC. Moreover, accumulating evidence indicates that noncoding regions of the genome, especially long noncoding RNAs (lncRNAs), are critically involved in the initiation and progression of EC [5].

lncRNAs play a crucial role in the pathogenesis of various diseases by modulating and impacting key cellular processes, including differentiation, proliferation, apoptosis, and metastasis [6]. Nevertheless, they do not operate independently; their regulatory influence is exerted through interactions with downstream target genes [7].

LINC00839 is a lncRNA with five exons at chromosomal region 10q11.21. It is involved in non-cancerous diseases such as osteoarthritis and lung damage. Additionally, differential expression of LINC00839 has been observed in breast and lung cancers. LINC00839 may be involved in tumor development and progression by affecting various signaling pathways [8]. Consequently, LINC00839 is considered a promising biomarker and a potential therapeutic target.

LINC01605 is situated on chromosome 8p11.23 and has been implicated in the pathogenesis of multiple cancer types. Dysregulated expression of LINC01605 has been documented in colorectal, pancreatic, breast, and squamous cell carcinomas. Moreover, evidence suggests that LINC01605 influences cancer cell behavior through diverse signaling pathways [9].

To date, no studies have investigated the role of LINC00839 and LINC01605 in EC, leaving their potential contribution to its pathogenesis unexplored. This study evaluates the LINC00839 and LINC01605 genes in EC, investigating their expression patterns, associated interactions with miRNAs and mRNAs, and potential roles in the disease's pathology and progression.

## 2 Materials and methods

### 2.1 The expression of LINC00839 and LINC01605 was investigated using GEPIA

Gene Expression Profile Interactive Analysis (GEPIA) (http://gepia.cancer-pku.cn/) was used to analyze the expression patterns of LINC00839 and LINC01605 in EC and to examine their differential expression across various stages of EC. This database is designed based on the analysis of cancer data from The Cancer Genome Atlas (TCGA) and Genotype-Tissue Expression (GTEx) databases [10]. In addition, the correlation between the expression levels of LINC00839 and LINC01605 in EC was retrieved from the GEPIA database.

### 2.2 Pan-cancer expression analysis

Utilizing the GEPIA database and applying the ∣log_2_FC| ≥ 1 and p-value <0.05 criterion, the expression profiles of the LINC00839 and LINC01605 genes across various cancer types were systematically analyzed.

### 2.3 Predicting interactions between lncRNAs-miRNAs and miRNA-mRNA

The interactions between LINC00839, LINC01605, and their target miRNAs were predicted using the DIANA-LncBase v3 online tool (https://diana.ece.uth.gr/lncbasev3) [11]. Subsequently, shared miRNAs were identified among those interacting with both lncRNAs. Next, mRNAs associated with these miRNAs were retrieved using the multiMiR package in R software. In this package, interactions between miRNAs and mRNAs are predicted and validated based on various databases, from which we selected interactions available in validated databases, including TarBase and miRTarBase [12]. Finally, shared mRNAs interacting with the identified miRNAs were determined.

### 2.4 Survival analysis of LINC00839 and LINC01605 in EC

The prognostic relevance of LINC00839 and LINC01605 in EC was evaluated using Overall Survival and Disease-Free Survival data retrieved from the GEPIA database, with time units standardized to months.

### 2.5 Constructing the interaction network between lncRNA-miRNA-mRNA

The lncRNA, miRNA, and mRNA interaction network was constructed using Cytoscape software. This network incorporated the previously identified miRNAs and mRNAs to visualize their interactions based on the competing endogenous RNA (ceRNA) framework. In this network, nodes represented biological entities, including lncRNAs, miRNAs, and mRNAs, while edges indicated their molecular relationships.

### 2.6 Protein-protein interaction (PPI) network, functional Classification, and pathway enrichment of mRNAs

The protein–protein interaction (PPI) network of the predicted mRNAs targeted by the identified miRNAs was constructed using the GeneMANIA plugin in Cytoscape [13].

Enriched pathway and function information for predicted mRNAs associated with the miRNAs was performed by Gene Ontology (GO) and Kyoto Encyclopedia of Genes and Genomes (KEGG) enrichment using the clusterProfiler package in R software [14]. Significantly enriched GO terms and KEGG pathways (p-value <0.05) were selected.

### 2.7 Analysis of gene-disease association

DisGeNET (https://disgenet.com) is a comprehensive platform for analyzing gene-disease associations. It encompasses various connections between genes and diseases across diverse pathological conditions [15]. We selected 10 diseases that were associated with the LINC00839 and LINC01605 genes.

### 2.8 Drug target analysis

Drugs targeting the common mRNAs that interact with miRNAs were identified using DrugBank (https://go.drugbank.com/). Only drugs that have successfully passed the approval process were reported from these.

### 2.9 Patient sample Collection

After approval by a pathologist, 18 paired samples of EC tissues and adjacent non-tumor margins were obtained from the National Tumor Bank of Iran, which the Cancer Institute at Tehran University of Medical Sciences maintains. Exclusion criteria for the study included prior chemotherapy, radiotherapy, tumor recurrence, and any history of malignancy in the esophagus or other organs. The study was conducted in accordance with the Declaration of Helsinki, and all procedures received ethical approval from the Ethics Committee of Hamadan University of Medical Sciences (Ethical Code: IR.UMSHA.REC.1403.700).

### 2.10 Total RNA Extraction, cDNA Synthesis, and RT-qPCR

Total RNA was extracted from the prepared tissues using RNX-Plus solution (SinaClon, Iran). After confirming the purity and integrity of the extracted RNAs using Nanodrop (Thermo Fisher Scientific Inc., USA) and 1 % agarose gel electrophoresis, 1 μg of total RNAs was reverse transcribed into first-strand cDNAs (Pars Tous, Iran). Subsequently, relative gene expression levels were determined using the Roche Light Cycler 96 system (Roche Life Science, Germany) with RealQ Plus 2x Master Mix Green (Ampliqon, Denmark). The relative expression was normalized to β-actin and calculated using the 2^−ΔΔCt^ method [16]. The primer sequences are presented in Table 1.

**Table 1:** The primer sequences.

Gene			
LINC00839	Human	Forward	GAACCTGTGGCATCCATCTC
		Reverse	CTCCAGCAACCCCTCAACC
LINC01605	Human	Forward	TGTGTGACAGAATGGGACCTG
		Reverse	TCGGCTGTTTGTAACGGGA
β-actin	Human	Forward	GAGCCTCGCCTTTGCCGATCC
		Reverse	ACATGCCGGAGCCGTTGTCG

### 2.11 Statistical analysis

Statistical analyses were performed using GraphPad Prism 8. The data are presented as mean ± standard error of the mean (SEM), with statistical significance defined as p-values <0.05. Depending on the distribution pattern of the data, either an independent sample *t*-test was used for normally distributed data or a Mann-Whitney *U* test for non-normally distributed data. Receiver Operating Characteristic (ROC) curves were constructed to assess the genes' diagnostic potential in EC.

## 3 Results

### 3.1 Expression of LINC00839 and LINC01605 genes in EC

**Fig. 1:**
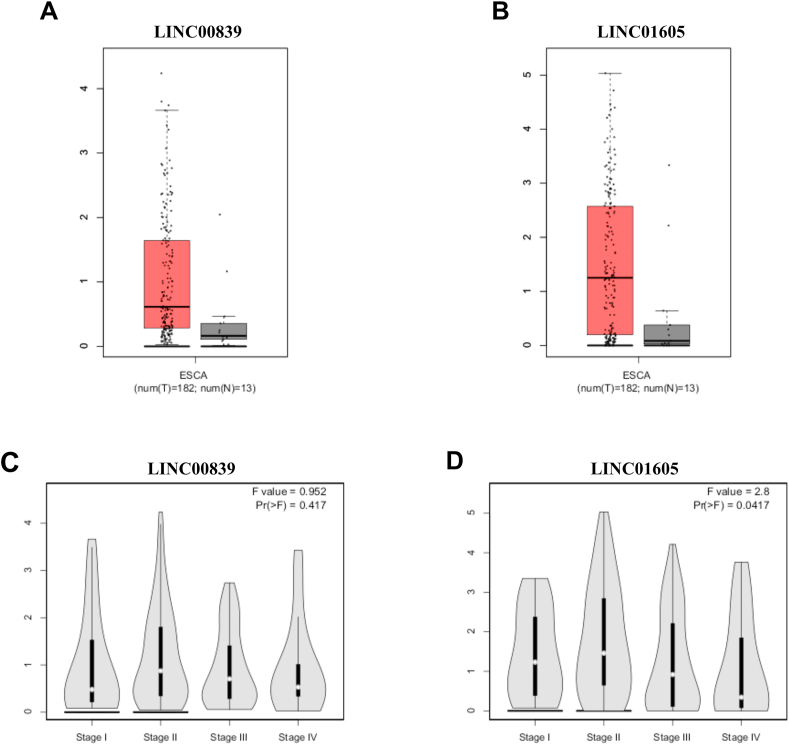
Boxplots and stage plots were obtained from the GEPIA database and are related to LINC00839 and LINC01605 in EC. (A) LINC00839 expression is non-significantly increased in EC. (B) LINC01605 expression is significantly increased in EC. The red and gray boxes represent cancer and normal tissues, respectively. (C) LINC00839 expression is unchanged in different stages of EC. (D) LINC01605 expression is decreased in the higher stages of EC. ESCA: Esophageal carcinoma.

**Fig. 2:**
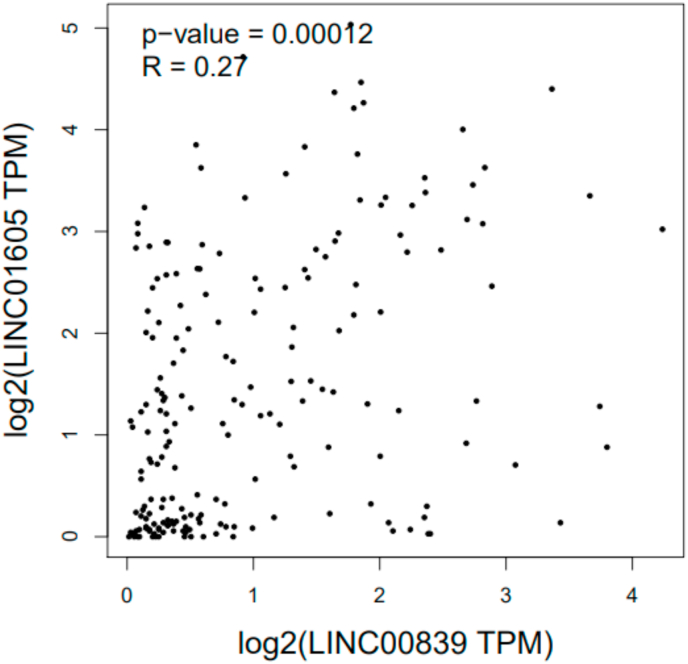
Correlation between expression of LINC00839 and LINC01605 genes in EC. The expression of LINC00839 and LINC01605 genes in EC has a significant positive correlation (R = 0.27, p-value<0.05).

Analysis of the GEPIA database revealed a non-significant upregulation of LINC00839 in EC (Fig. 1 A), with no significant variation across disease stages (p-value>0.05, Fig. 1 C). LINC01605 expression was significantly elevated in EC (Fig. 1 B) but was lower in stages 3–4 compared to stages 1–2 (p-value<0.05, Fig. 1 D). Moreover, a significant positive correlation was identified between the expression levels of the LINC00839 and LINC01605 genes in EC (Fig. 2). These results highlight the significance of LINC01605 in EC tumors, especially in high-stage groups, and its correlation with LINC00839.

### 3.2 Alteration of LINC00839 and LINC01605 expression in pan-cancers

**Fig. 3:**
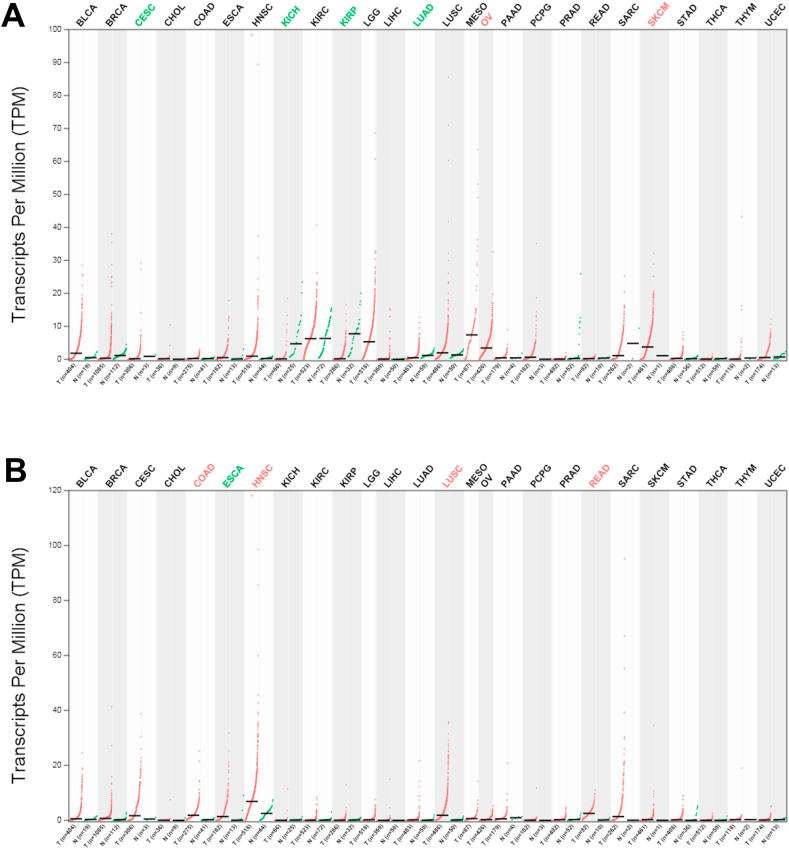
Expression of LINC00839 and LINC01605 genes in various cancers according to the GEPIA database. (A) LINC00839 expression differs in OV, SKCM, CESC, KICH, KIRP, and LUAD. (B) LINC01605 expression is increased in COAD, ESCA, HNSCC, LUAD, and READ cancers. BLCA: Bladder Urothelial Carcinoma, BRCA: Breast invasive carcinoma, CESC: Cervical squamous cell carcinoma and endocervical adenocarcinoma, CHOL: Cholangiocarcinoma, COAD: Colon adenocarcinoma, HNSC: Head and Neck squamous cell carcinoma, KICH: Kidney Chromophobe, KIRC: Kidney renal clear cell carcinoma, KIRP: Kidney renal papillary cell carcinoma, LGG: Brain Lower Grade Glioma, LIHC: Liver hepatocellular carcinoma, LUAD: Lung adenocarcinoma, LUSC: Lung squamous cell carcinoma, MESO: Mesothelioma, OV: Ovarian serous cystadenocarcinoma, PAAD: Pancreatic adenocarcinoma, PCPG: Pheochromocytoma and Paraganglioma, PRAD: Prostate adenocarcinoma, READ: Rectum adenocarcinoma, SARC: Sarcoma, SKCM: Skin Cutaneous Melanoma, STAD: Stomach adenocarcinoma, THCA: Thyroid carcinoma, THYM: Thymoma, and UCEC: Uterine Corpus Endometrial Carcinoma.

Increased LINC00839 gene expression was found in ovarian (OV) and Skin Cutaneous Melanoma (SKCM) cancers. In contrast, its expression was downregulated in Cervical Squamous Cell Carcinoma and Endocervical Adenocarcinoma (CESC), Kidney Chromophobe (KICH), Kidney Renal Clear Cell Carcinoma (KIRP), and Lung Adenocarcinoma (LUAD) (Fig. 3 A). Although LINC00839 showed an upregulation in Esophageal Carcinoma (ESCA), this elevation was not statistically significant.

The expression of the LINC01605 gene was found to be upregulated in several cancers, including Colon Adenocarcinoma (COAD), ESCA, Head and Neck Squamous Cell Carcinoma (HNSCC), Lung Adenocarcinoma (LUAD), and Rectum Adenocarcinoma (READ) (Fig. 3 B).

### 3.3 Survival analysis of LINC00839 and LINC01605 genes

**Fig. 4:**
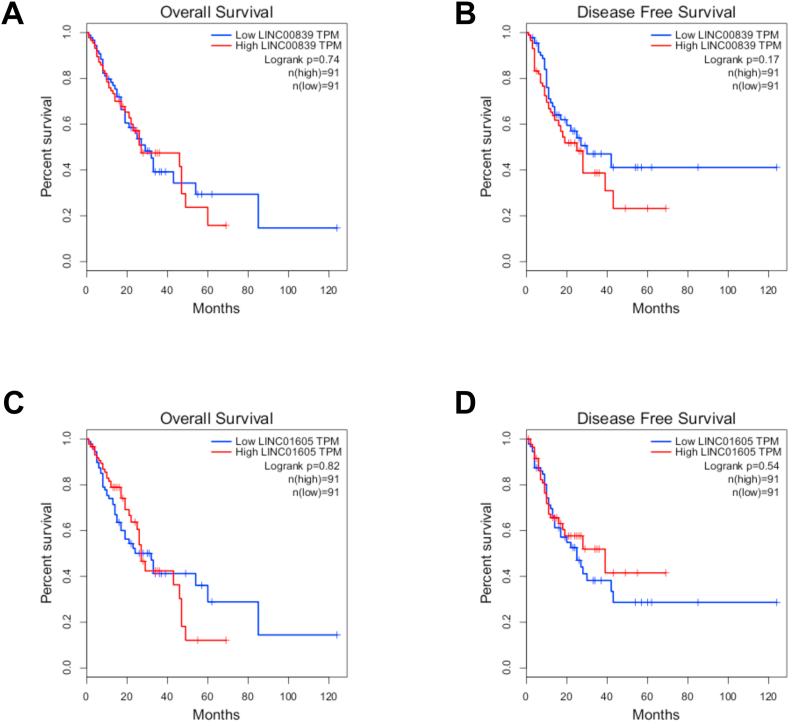
The relationship between genes and patients' overall survival and disease-free survival outcomes. Prognostic curves of LINC00839 and LINC01605 genes showed an insignificant survival rate (P > 0.05). The red lines represent patients with high gene expression, and the blue lines represent low gene expression. HR: hazard ratio.

Overall survival and disease-free survival analyses were conducted using the GEPIA database, stratifying patients into high and low-expression groups based on median gene expression levels. Neither LINC00839 nor LINC01605 expression was significantly associated with EC patient survival (Log-rank p > 0.05, Fig. 4). However, higher expression of LINC00839 demonstrated a declining trend in disease-free survival among EC patients.

### 3.4 Prediction of lncRNA-miRNA and miRNA-mRNA interactions

**Fig. 5:**
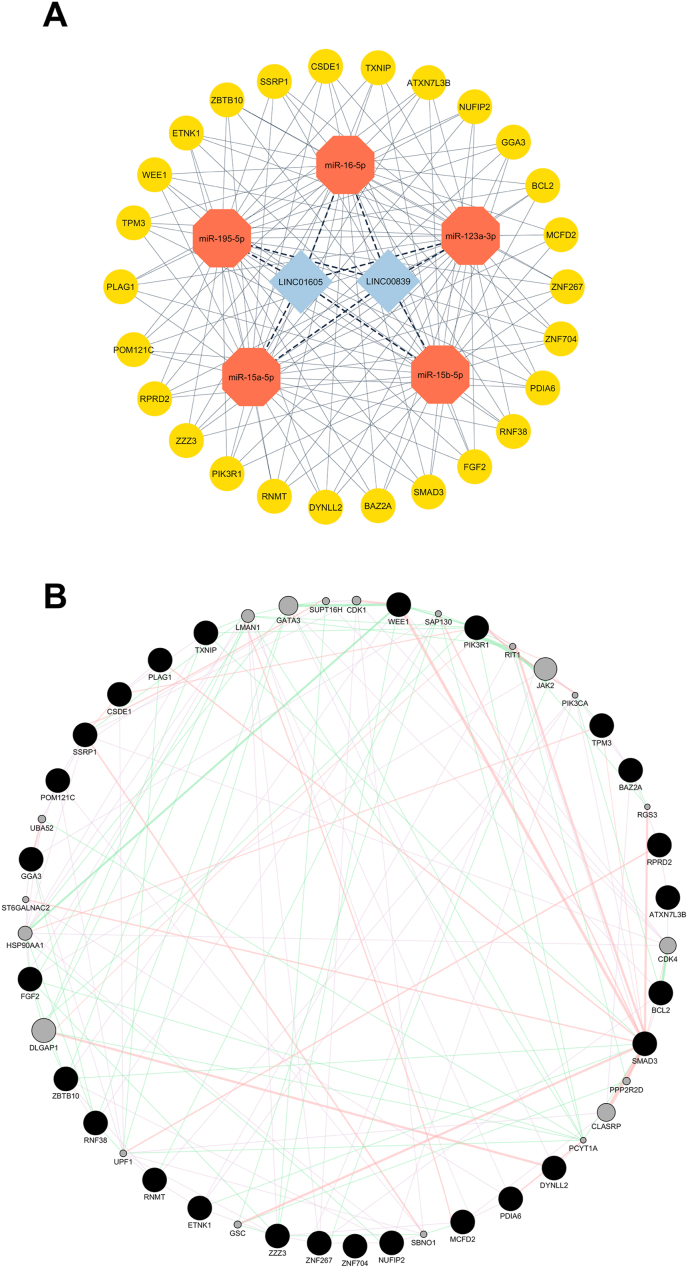
ceRNA and PPI network. (A) Interaction network between lncRNAs, miRNAs, and mRNAs. (B) PPI network obtained for shared mRNAs from the GeneMANIA plugin of Cytoscape software.

The DIANA-LncBase online tool revealed that the miR-16-5p, miR-195-5p, miR-123a-3p, miR-15a-5p, and miR-15b-5p commonly interact with both lncRNAs (LINC00839 and LINC01605). The multiMiR package predicted 26 target mRNAs of these miRNAs, including ATXN7L3B, BAZ2A, BCL2, CSDE1, DYNLL2, ETNK1, FGF2, GGA3, MCFD2, NUFIP2, PDIA6, PIK3R1, PLAG1, POM121C, RNF38, RNMT, RPRD2, SMAD3, SSRP1, TPM3, TXNIP, WEE1, ZBTB10, ZNF267, ZNF704, and ZZZ3. A ceRNA network comprising two lncRNAs, five miRNAs, and 26 mRNAs was visualized using Cytoscape software, as shown in Fig. 5 A. Additionally, a protein–protein interaction (PPI) network of the 26 target mRNAs was constructed using the GeneMANIA plugin within Cytoscape, as illustrated in Fig. 5 B.

### 3.5 GO and KEGG enrichment pathway analyses of genes

**Fig. 6:**
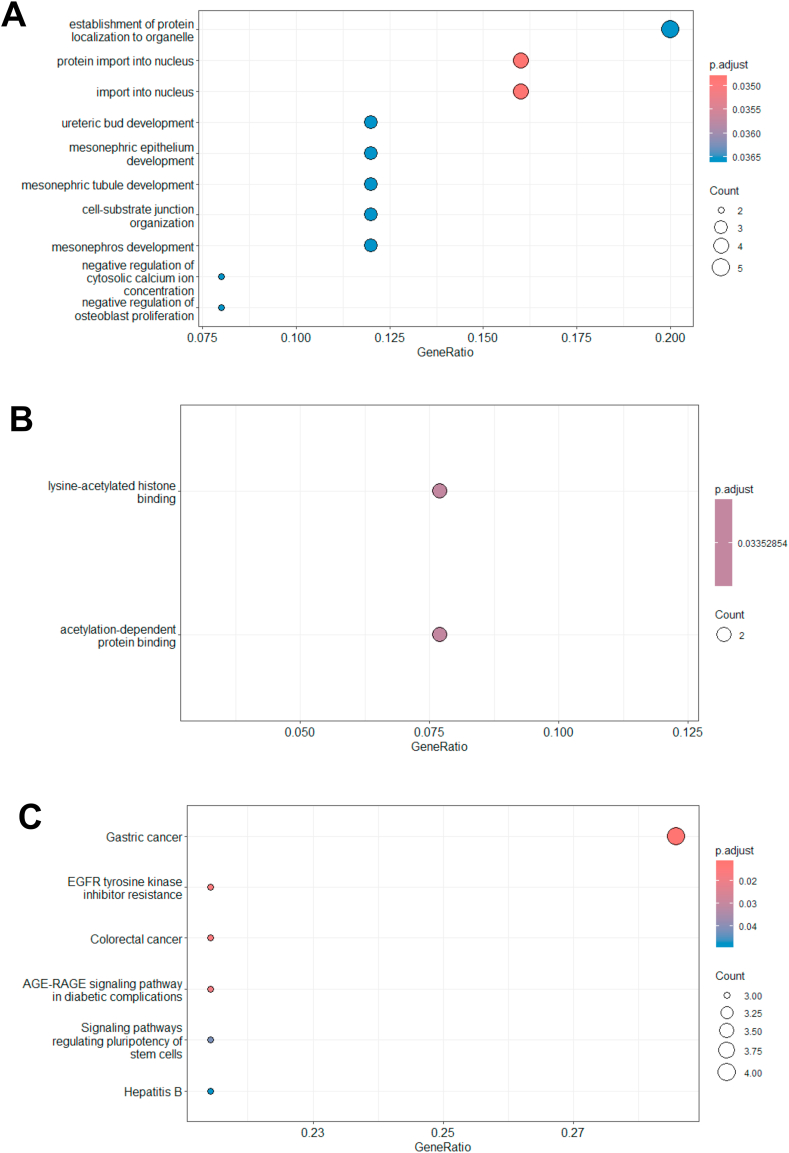
Dot plot of Gene Ontology (GO) and Kyoto Encyclopedia of Genes and Genomes (KEGG) analyses. Each Dot plot indicates significantly enriched terms in GO categories and KEGG results, including (A) biological process (BP), (B) molecular function (MF), and (C) KEGG. The dot plots at the chart's top are more significant than those at the bottom.

GO and KEGG enrichment analyses were performed to examine the biological functions and signaling pathways associated with the common mRNAs interacting with miRNAs. Within the Biological Process (BP) category, the most enriched pathways included protein localization to organelles, protein import into the nucleus, and nuclear import mechanisms (Fig. 6 A). The only significant pathways within the Molecular Function (MF) category included lysine-acetylated histone binding and acetylation-dependent protein binding (Fig. 6 B). KEGG analysis highlighted pathways such as gastric cancer, EGFR-tyrosine kinase inhibitor resistance, and colorectal cancer (Fig. 6 C). Conversely, no significant pathways were identified in the Cellular Compartment (CC) category.

### 3.6 Gene-diseases association

Table 2 documents diseases associated with the LINC00839 and LINC01605 genes. The LINC00839 gene is most associated with nasopharyngeal carcinoma and neuroblastoma, while the LINC01605 gene is most associated with colorectal carcinoma and neoplasm.

**Table 2:** Diseases associated with LINC00839 and LINC01605 genes were obtained using the DisGeNET Platform.

LINC00839	Score	LINC01605	Score
Nasopharyngeal carcinoma	0.2	Colorectal Carcinoma	0.2
Neuroblastoma	0.2	Colorectal Neoplasms	0.2
Nasopharyngeal carcinoma	0.2	Nasopharyngeal carcinoma	0.15
Neuroblastoma	0.2	Laryngeal Squamous Cell Carcinoma	0.1
Carcinogenesis	0.1	Conventional Renal Cell Carcinoma	0.1
Adenocarcinoma of lung	0.1	Malignant neoplasm of ovary	0.1
Glioblastoma Multiforme	0.1	Bladder Neoplasm	0.1
Carcinoma of bladder	0.1	Carcinoma of bladder	0.1
Malignant neoplasm of urinary bladder	0.1	Malignant tumor of colon	0.1
Tumor Progression	0.1	Cervical Cancer	0.1

### 3.7 Drug target analysis

Among the shared mRNAs, FGF2, MCFD2, and PIK3R1 are the only ones for which drugs have received approval for targeting them. Heparin, Pentosan polysulfate, and Sucralfate target FGF2. MCFD2 is exclusively targeted by Moroctocog alfa, which has anti-hemorrhagic properties. Alpelisib is the only approved drug targeting PIK3R1 (Table 3).

**Table 3:** Approved drugs targeting shared mRNAs according to DrugBank.

Genes	Drug name	Drug group	Drug category	Drug Targets	Drug background
FGF2	Heparin	Approved, Investigational	Anticoagulants	P-selectin, FGFR-4, FGF4, FGF19, FGFR1, FGF1, FGFR2, PF4, HGF	A type of blood thinner used to prevent blood clots in a variety of medical conditions
FGF2	Pentosan polysulfate	Approved, Investigational	Glycosaminoglycan	FGF1, FGF4	Pentosan polysulfate is a sulfated pentosyl polysaccharide used to treat bladder pain and discomfort due to interstitial cystitis
FGF2	Sucralfate	Approved, Investigational	Aluminum Complex	PGA5, Pro-EGF	A medication used to treat ulcers in the stomach and intestines and to prevent these ulcers from coming back in the future
MCFD2	Moroctocog alfa	Approved	Hemostatics	Coagulation factor X, Coagulation factor IX, vWF	A medication used to treat bleeding disorders and stop bleeding
PIK3R1	Alpelisib	Approved, Investigational	phosphatidylinositol 3-kinase inhibitor	ERs, PIK3CA	Alpelisib is a phosphatidylinositol 3-kinase (PI3K) inhibitor with potent antitumor activity

### 3.8 Basic Characteristics of patients

The majority of patients with EC were women. Most cases were diagnosed at stages II and III, with the predominant tumor grade being grade 2. Additionally, lymph node metastasis was common among the patients, and most were classified as T3 based on pathological staging (Table 4).

**Table 4:** Clinicopathological parameters of EC patients.

Parameters	Gender		Clinical stage			Grade			Pathological T			Lymph Nodes	
Types	*Male Female*		*II III IV*			*1 2 3*			*T2 T3 T4*			*N0 N1*	
Number of patients	*7*	*11*	*9*	*8*	*1*	*5*	*11*	*2*	*5*	*12*	*1*	*6*	*12*

### 3.9 Expression of LINC00839 and LINC01605 genes in patients with EC

**Fig. 7:**
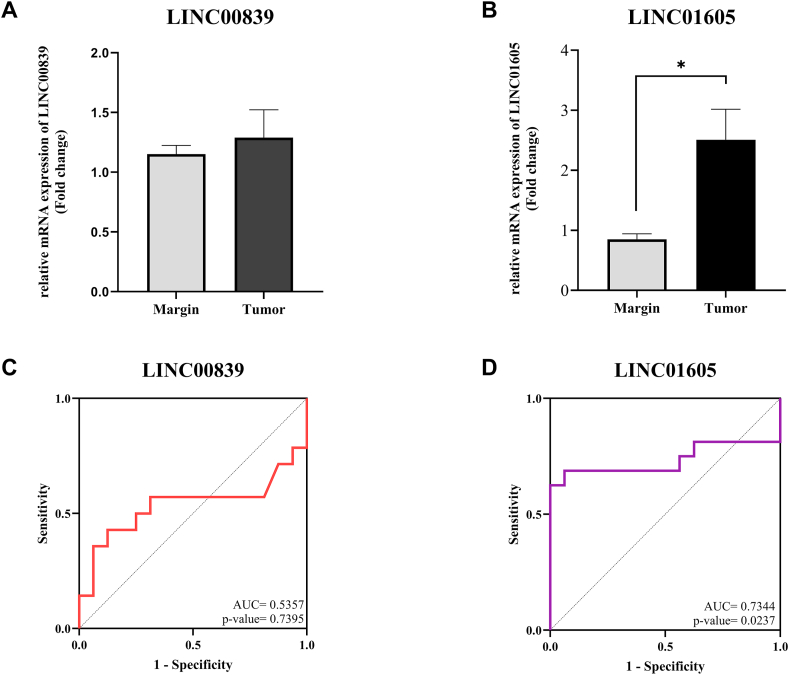
Gene expression and ROC curve of LINC00839 and LINC01605. (A) LINC00839 gene expression was non-significantly increased in cancer samples. (B) The expression of the LINC01605 gene was significantly increased in the EC samples. (C–D) LINC01605 gene has good diagnostic value in EC patients, unlike LINC00839.

RT-qPCR validation in 18 paired EC and adjacent non-tumor tissues confirmed the GEPIA findings. Consistent with bioinformatics data, LINC00839 showed a non-significant upregulation in EC tissues (logFC = 1.28, p-value>0.05, Fig. 7 A). In contrast, LINC01605 gene expression was significantly increased in EC tissues (logFC = 2.5, p-value<0.05, Fig. 7 B).

### 3.10 ROC curve analysis

ROC curve analysis was used to determine the diagnostic value of LINC00839 and LINC01605 in EC. The LINC00839 gene had an area under the curve (AUC) value of 0.5357 (95 % CI: 0.3027 to 0.7687) and a p-value>0.05 (Fig. 7 C). In contrast, LINC01605 showed a good diagnostic value with an AUC of 0.7344 (95 % CI: 0.5339 to 0.9348) and a p-value< 0.05 (Fig. 7 D).

## 4 Discussion

Recently, increased research on lncRNAs has highlighted their role in cancer [17]. However, studies specifically focusing on lncRNAs in EC remain limited. Emerging evidence suggests that lncRNAs contribute to both the initiation and progression of esophageal malignancies, functioning either as oncogenic drivers or tumor suppressors depending on their molecular context and regulatory targets [18]. For example, studies on the lncRNA MALAT1 have revealed its oncogenic function in EC, where it promotes cell cycle progression and suppresses apoptosis. Additionally, MALAT1 enhances tumor activity by facilitating the dephosphorylation of the ATM protein [19]. Conversely, the lncRNA NEF has been identified as a tumor suppressor in EC. NEF inhibits the proliferation, migration, and invasion of esophageal tumor cells by modulating the Wnt/β-catenin signaling pathway [20].

This study used bioinformatic tools and experimental validation to explore the expression and potential functions of LINC00839 and LINC01605 in EC.

Our bioinformatic analyses and RT-qPCR results from EC tissue samples show that LINC00839 was increased in EC samples, but this increase was insignificant. In a previous study, we found that LINC00839 acts as an oncogene in oral squamous cell carcinoma (OSCC) through the miR-195-5p/cyclin E1 axis [21]. In contrast to the current study's findings in EC, LINC00839 showed a significant AUC in OSCC, indicating its potential as a diagnostic biomarker in that context [21]. In another study, Zhang and colleagues found that LINC00839 promotes nasopharyngeal carcinoma invasion through the miR-454-3p/c-Met axis [22]. These findings underscore LINC00839's role in various cancers through distinct pathways.

The present study also examined LINC01605 expression. Consistent with the bioinformatic predictions, RT-qPCR analysis confirmed a significant upregulation of LINC01605 in EC tissue samples. Furthermore, LINC01605 demonstrated favorable AUC values in the ROC curve, indicating its potential utility as a biomarker and a promising candidate for therapeutic intervention. S–S Hu et al. identified that LINC01605 could act as an oncogene in colorectal cancer. LINC01605 is notably overexpressed in colorectal cancer tissues. Furthermore, LINC01605 increased cancer cell proliferation, migration, invasion, and decreased apoptosis through the miR-3960/SOX11 axis [23]. In nasopharyngeal carcinoma, LINC01605 similarly acts as an oncogene by enhancing cell proliferation and inhibiting apoptosis. It facilitates the nuclear translocation of p65 and activates the NF-κB signaling pathway by regulating IKBKB through sponging miR-942-5p [24]. GEPIA database analysis revealed a downregulation of LINC01605 expression in patients with stage 3–4 EC. Conversely, in colorectal cancer, LINC01605 is upregulated in cases with lymph node involvement, distant metastases, and advanced tumor stages [25]. This difference likely reflects the cancer-type-specific role of LINC01605, as lncRNAs can behave differently depending on the tumor context and gene regulation patterns.

Kaplan–Meier survival analyses of LINC00839 and LINC01605, based on data retrieved from the GEPIA database, revealed no statistically significant association between their expression levels and overall or disease-free survival in patients with EC. This lack of significance may be attributed to several confounding factors, including tumor heterogeneity, patient comorbidities, and underlying molecular variations [26].

According to the ceRNA hypothesis, different types of RNAs, such as lncRNAs, mRNAs, and circRNAs, communicate through shared miRNA response elements and competitively bind to miRNAs, thereby modulating each other's expression [27]. Based on DIANA-LncBase database analysis, our bioinformatic results revealed that miR-16-5p, miR-195-5p, miR-123a-3p, miR-15a-5p, and miR-15b-5p jointly interact with both LINC00839 and LINC01605. These miRNAs may be involved in the pathogenesis of colorectal [28], cervical [29], bladder [30], and nasopharyngeal cancers [31]. Li et al. reported that decreased serum and tissue levels of miR-15a in ESCC are significantly associated with advanced tumor stage, poor differentiation, and reduced overall survival, identifying miR-15a as an independent prognostic biomarker for ESCC [32]. Additionally, increased miR-195-5p expression in EC has been shown to reduce cell viability, colony-forming capacity, and invasive potential [33]. Consistent with the role reported for these miRNAs in most previous studies, the miRNAs identified in our analysis as potential downstream targets of LINC00839 and LINC01605 may function as tumor suppressors in EC.

Based on multiMiR package analysis in R software and DrugBank database results, Fibroblast Growth Factor 2 (FGF2), Multiple Coagulation Factor Deficiency Protein 2 (MCFD2), and Phosphoinositide 3 Kinase Regulatory Subunit 1 (PIK3R1) were identified as druggable downstream mRNA targets of the selected miRNAs. FGF2 is overexpressed in EC tissues and is also associated with tumor size, gender, and lymph metastasis in patients with EC [34]. Reduced expression of the PIK3R1 gene has been reported in EC cell lines. Furthermore, hsa_circ_0087104 enhances PIK3R1 expression in EC by suppressing miR-542-3p, resulting in decreased migration and invasion of cancer cells [35]. The MCFD2 gene is upregulated in OSCC cell lines. Increased expression of this gene is associated with increased metastasis and decreased cell adhesion. These findings suggest an important role of MCFD2 in OSCC metastasis [36]. Based on the ceRNA hypothesis, our study identified putative downstream mRNAs regulated by miRNAs, such as FGF2, MCFD2, and PIK3R1, which are often reported to exert oncogenic functions in various cancers, consistent with the findings of most previous studies.

This study integrates bioinformatics predictions and experimental validation to offer preliminary insights into the potential roles of LINC00839 and LINC01605 in EC. Interestingly, the expression of LINC00839 and LINC01605 molecules was positively correlated in EC. It also constructs a ceRNA-based network to predict their downstream miRNAs and mRNAs.

Building on the findings of this study, future research should focus on validating the diagnostic and prognostic potential of LINC00839 and LINC01605 in EC through large-scale cohort studies conducted across multiple clinical centers. Such investigations would help establish their clinical relevance and generalizability. Moreover, the therapeutic applicability of these lncRNAs warrants exploration through the design and evaluation of small interfering RNAs targeting their expression in prospective trials. These approaches may offer promising avenues for the development of RNA-based diagnostic tools and targeted therapies in EC.

### 4.1 Limitations

To further elucidate the biological significance of LINC00839 and LINC01605 in EC, future research should incorporate functional assays such as knockdown and overexpression studies. These approaches will help clarify the mechanistic roles of these lncRNAs in tumor progression. Moreover, investigations involving larger patient cohorts and blood-derived samples are essential to validate their diagnostic and prognostic utility and to better understand their contributions to the development and progression of EC.

## 5 Conclusion

The study explored the expression patterns of LINC00839 and LINC01605 in EC using bioinformatic and experimental approaches. While LINC00839 showed an insignificant increase, LINC01605 was significantly upregulated and exhibited good AUC values, suggesting its potential as a biomarker. Additionally, miR-16-5p, miR-195-5p, miR-123a-3p, miR-15a-5p, and miR-15b-5p jointly interacted with both lncRNAs, highlighting a shared regulatory network involved in tumor progression.

## CRediT authorship contribution statement

**Mahdi Bahmani:** Writing – original draft, Investigation, Formal analysis. **Ashkan Kalantary-Charvadeh:** Writing – review & editing, Methodology, Data curation. **Morvarid Hamrahjoo:** Software, Methodology. **Nasrin Ziamajidi:** Methodology, Formal analysis. **Roghayeh Abbasalipourkabir:** Writing – review & editing, Funding acquisition, Data curation. **Shayan Marhamati:** Writing – original draft, Supervision, Project administration, Conceptualization.

## Ethics approval and informed consent

The study was approved by the local ethics committee at Hamadan University of Medical Sciences (IR.UMSHA.REC.1403.700). The Declaration of Helsinki has carried out the work described for experiments involving humans. Informed consent was obtained for experimentation with human samples, and the privacy rights of human subjects have been observed.

## Funding

The study was funded by the Vice-Chancellor for Research and Technology, 10.13039/501100004697Hamadan University of Medical Sciences (No. 140310048774).

## Declaration of competing interest

The authors declare that they have no conflict of interest.

## Data Availability

Data will be made available on request.
